# Parental knowledge and practice on antibiotic use for upper respiratory tract infections in children, in Aksum town health institutions, Northern Ethiopia: a cross-sectional study

**DOI:** 10.11604/pamj.2020.35.142.17848

**Published:** 2020-04-29

**Authors:** Teklay Zeru, Hagos Berihu, Gerezgiher Buruh, Haftom Gebrehiwot, Mebrahtom Zeru

**Affiliations:** 1 School of Nursing, College of Health Sciences, Aksum University, Aksum, Ethiopia; 2 School of Nursing, College of Health Sciences, Mekelle University, Mekelle, Ethiopia; 3 Department of Biomedical Science, College of Health Sciences, Adigrat University, Adigrat, Ethiopia

**Keywords:** Knowledge of parents on antibiotics, practice of parents on antibiotic, URTI, Aksum town

## Abstract

**Introduction:**

worldwide, antibiotics are the most commonly prescribed and abused drugs for upper respiratory tract infections. Acute upper respiratory infections are common in children who attend childcare and preventing transmission of disease in health setting depends on actions by parents and staff. Therefore the objective of this study is to assess the parental knowledge and practice on antibiotic use for upper respiratory tract infections in children, in Aksum town health institutions, northern Ethiopia, 2018.

**Methods:**

a facility-based cross-sectional study design was adopted involving 384 parents of children visited governmental health facilities in Aksum town from February to March, 2018. Respondents were selected based on the proportion of nurses in the health facilities. SPSS version 22 was applied for data entry and analysis.

**Results:**

the total number of questionnaires was 384 resulting in a 100% response rate. Almost half of the parents had poor knowledge of the use of antibiotics in children for URTIs 183(47.7%), followed by 156(40.6%) moderate knowledge and 45(11.7%) good knowledge. Practices regarding antibiotic use in children with URTI varied. Only 12.8% of the parents did not always follow the doctors´ advice regarding antibiotic use. In this study has reported many areas in which parental awareness on antibiotic use for acute URTI is considered inadequate, consequently inappropriate knowledge and practices.

**Conclusion:**

nearly half of the parents attending the physicians for their children with URTI expected to get antibiotics.

## Introduction

Worldwide, children take large amounts of antibiotics. Majority of infections were, upper respiratory tract infections (URTI) [1], upper respiratory tract infections (URTIs) are most common among children [2]. Acute respiratory infections and diarrheal diseases are the leading causes of childhood death, resulting in 25-33% of all mortality in children in developing countries. Globally, antibiotics are the most commonly prescribed and abused drugs for upper respiratory tract infections (URTIs) [3, 4]. The common includes: common cold, viral or bacterial tonsillopharyngitis, Otitis media, acute rhinosinusitis and croup are examined specifically [5]. However, a reduced trend in antibiotic prescribing over the years, over-prescription of antibiotics [6]. Though it appears that antibiotic malpractice is not driven by parental pressure, pediatricians´ view implies that medical doctors feel that most of the parents wish for antibiotic application for their children´s URTIs [7]. Presence of to daycare centers upward the susceptibility of upper respiratory symptoms and problem in 3- to 5-year-old children [8]. Antibiotic prescribing for patients with common colds, upper respiratory tract infections, sinusitis and bronchitis: a countrywide study of the hospital-based emergency department [9].

All prescribers and soled cited patient demands for antibiotics as the main purpose of prescribing antibiotics for URTI [10-12]. In the 18-month study time, the parents of all children admitted through the day surgical unit were confused about whether their child had a URI [13]. The deficit of efficacy and minimized complication rates, antibiotic management of children with URTI is not supported by the latest report from randomized trials [14]. Acute upper respiratory infections are a major problem in children who attend childcare and preventing transmission of disease in this setting depends on caring habits of by childcare staff [15]. Though children with acute and latest URIs are at high risk for respiratory complications, these results suggest that most of these children can undergo elective procedures without a significant increment in adverse anesthetic effect [16]. There has been a great reduction in community antibiotic prescribing related to the specific diagnoses of lower respiratory tract infection, tonsillitis, pharyngitis and acute Otitis media. Generally prescribing is now increasing similarly, but associated with non-specific upper respiratory tract infection identifies [17].

URTI were documented over the previous one year and at that time reported as: history of acute Otitis media and recent family history of respiratory infection were also obtained [1]. Physicians to be concerned about possible bacterial infection in children are associated with antibiotic use for pediatric URI [18]. On the current report, as long as patients have clear and specific information about when to use antibiotics and when to return for reassessment, delayed prescribing of antibiotics for upper respiratory tract infection is probably as safe or safer than other options, affordable and is acceptable to clients [19]. Therefore, the value of parental knowledge to nurses who deliver the majority of care to chronical patients is unquestionable [20] and there is a need to support and educate nurses for the parents on parental awareness of antibiotics [21], so, the first step in developing a strategy to support and educate nurses about parental knowledge of antibiotics is to assess their current practice as there is limited research [22]. Let's conclude that this study aims to assess nurse´s practice towards parental knowledge on antibiotics for URTIs among nurses working in Aksum town.

## Methods

The study was conducted in Aksum town which is located 1024km and 241km far from the capital city of Ethiopia, Addis Ababa and the capital city of Tigray, Mekelle, respectively. The study period was from February to June 2018. The institution-based cross-sectional study design was used to address the objective parental knowledge and practice on antibiotic use for upper respiratory tract infections in children from parents. The data was collected by self-administered questionnaire to address parental knowledge and practice on antibiotic use. All parents of children bring to governmental health institution Aksum town. All sampled parents of those come with their child to governmental health institution Aksum town. All parents come to health institution with their child were included in the study but parents not available in the study area during data collection were excluded. The sample size will be calculated using a single population proportion formula based on the following assumptions:

n=(Zα/2)^2^p (1-p)/(d)^2^

where n = minimum sample size required for the study, d=margin of error = 0.05, Zα/2 value of standard normal distribution (Z = 1.96) with confidence interval of 95% and α is 0.05. P is taking by 50% because a similar study is not conducted in Ethiopia:

n = (Zα/2)^2^ p(1-p)/(d)^2^;

n = (1.96)^2^ 0.5(1-0.5)/(0.05)^2^ = 384.

To get the total sample we have used a systematic sampling technique from Aksum comprehensive specialized hospital, St. Marry Hospital, Aksum Health Center and Millennium Health Center every 2^nd^ parent of the visited child was taken from the governmental health facilities of Aksum town. But the first sample was selected by simple random sampling (lottery method). Data were collected by self-administered questionnaire using adopted from the literature of similar studies [7, 23-25]. To assure high quality of the data, the emphasis was given in designing data collection instrument and training on data collectors. Similarly, the questionnaire format was pretested before the actual data collection period to modify the tool. The collected data was checked for its completeness, consistency and accuracy before analysis. Data were analyzed and interpreted using SPSS version 22.

**List of operational definitions in this study:** good Knowledge = the parents must have a 75% knowledge on antibiotics to URTI treatment. Good knowledge aspect of practice =≥ 75% of total knowledge aspect of practice questions. Poor knowledge aspect of practice =< 75% of the total knowledge aspect of practice questions.

## Results

The total number of completed and returned questionnaires was 384 resulting in a 100% response rate. Of these 196(51.0%) were from St. Marry Hospital, 105(27.4%) represented Axum Health Center and 83 (21.6 %) were from the Millennium Health Center. Regardless of sex more female 224(58.3%) than male 160 (41.7%). The majority of respondents 235 (61.2%) were within the age of 21-29 years followed by 92(24%) were of 30-39 years. Most 359(93.5%) of the respondent ethnicity from Tigray. Majority of respondent were married 343(89.3%). A large number of participant was secondary school 154(40.1%) by educational level followed by primary school 73(19.0%) (Table 1).

**Table 1:** socio-demographic characteristics of nurses at Shire town health facilities in Tigray region, Ethiopia, June 2018

Characteristics		Frequency, n (%)
Gender	Male	160(41.7)
	Female	224(58.3)
age (years)	21-29	235(61.2)
	30-39	92(24.0)
	40-49	42(10.9)
	≥ 50	15(3.9)
Ethnicity	Tigray	359(93.5)
	Amara	23(6.0)
	Others	2(.5)
Marital status	Married	343(89.3)
	Widow	29(7.6)
	Divorced	12(3.1)
Educational level	No formal education	57(14.8)
	Primary	73(19.0)
	Secondary	154(40.1)
	Diploma	37(9.6)
	degree	63(16.4)
Occupation	Student	69(18.0)
	Housewife	109(28.4)
	Self-employed	103(26.8)
	Salaried employee	103(26.8)
Personal monthly income	0	131(34.1)
	<ETB 1000	73(19.0)
	ETB 1000-ETB 2000	81(21.1)
	ETB 2000-ETB 3000	56(14.6)
	ETB 3000-ETB 4000	19(4.9)
	> ETB 4000	24(6.3)
Household monthly income	0	0
	<ETB 1000	63(16.4)
	ETB 1000-ETB 2000	131(34.1)
	ETB 2000-ETB 3000	90(23.4)
	ETB 3000-ETB 4000	61(15.9)
	> ETB 4000	39(10.2)
Number of children	1	103(26.8)
	2	109(28.4)
	3	80(20.8)
	4	68(17.7)
	≥ 5	24(6.2)

**Parental knowledge on antibiotic use in children with URTIs:** the total score in the knowledge section was computed as the number of correct responses to the questions. Almost half of the parents had poor knowledge on the use of antibiotics in children for URTIs 183(47.7%), followed by 156(40.6%) moderate knowledge and 45(11.7%) good knowledge. The overall mean + SD of the respondents´ knowledge were 1.64+0.683. Out of the five questions, only three questions were answered correctly by more than half of the respondents, indicating poor Knowledge on the use of antibiotics in children with URTIs. Only 29.4% of the parents believed URTIs are of viral origin and do not require antibiotics to resolve the infection. Although 77.6% of the parents acknowledged that inappropriate use of antibiotic leads to bacterial resistance, more than half of the parents (55.5%) believed that flu-like symptoms resolved faster when antibiotics were given. Majority of parents 59.6% believes antibiotic use can prevent complications from Upper Respiratory Tract Infections (Table 2).

**Table 2:** parental knowledge on the use of antibiotics in children for URTIs in Aksum town governmental health institutions, June 2016(n=384)

Statements	Number of respondents, n (%)		
	Yes n	No n	Not sure
The antibiotic should be given to all children who develop a fever.	116(30.2	226(58.9)	42(10.9)
As most of the Upper Respiratory Tract Infections (e. g. cold, flu, sore throat, and ear infection) are of viral origin, antibiotics should not be given because they are self-limited.	113(29.4)	189(49.2)	82(21.4)
Children with flu-like symptoms get better faster when antibiotics are given.	213(55.5)	113(29.4)	58(15.1)
Inappropriate use of antibiotics reduces their efficacy and leads to bacterial resistance.	294(76.6)	67(17.4)	23(6.0)
Antibiotic use can prevent complications from Upper Respiratory Tract Infections.	229(59.6)	30(7.8)	125(32.6)

**Parental practices on antibiotic use in children with URTIs:** practices regarding antibiotic use in children with URTI varied. Only 12.8% of the parents did not always follow the doctors´ advice regarding antibiotic use. More than half 56.8% of the parents claimed they were sometimes informed by the doctors as to whether or not the antibiotics were necessary for the treatment of URTIs. The small number of parents (12.0%) believed that their doctors prescribed antibiotics because of their request. Only 1.6% of parents practiced how often do you completely follow all the medical doctor´s instructions and advice? Majority of parents 59.1% asked for the doctors to prescribe antibiotics (Table 3).

**Table 3:** parental practices on the use of antibiotics in children for URTIs (n=384)

Questions	Number of respondents, n (%)				
	Never	sometimes	often	Most of the times	Always
How often do you ask your medical doctor as to whether or not the prescription of antibiotics is necessary?	95(24.7)	131(34.1)	82(21.4)	27(7.0)	49(12.8)
How often does your medical doctor recommend antibiotics?	29(7.6)	218(56.8)	89(23.2)	24(6.3)	24(6.3)
How often do you ask directly for your medical doctor to prescribe antibiotics?	86(22.4)	151(39.3)	70(18.2)	31(8.1)	46(12.0)
How often do you completely follow all the medical doctor's instructions and advice?	6(1.6)	46(12.0)	100(26.0)	83(21.6)	149(38.8)
How often do you insist on your medical doctor's prescribing antibiotics as a precaution even if a diagnosis is not confirmed?	150(39.1)	84(21.9)	69(18.0)	55(14.3)	26(6.8)
How often does your medical doctor inform you about your child's disease and notify you whether it is necessary to receive antibiotics?	33(8.6)	83(21.6)	75(19.5)	85(22.1)	108(28.1)
How often do you think that your medical doctor prescribes antibiotics only because you asked him/her to?	227(59.1)	8(12.5)	70(18.2)	21(5.5)	18(4.7)

## Discussion

The overall parental knowledge on the use of antibiotics in children with URTIs was poor. Nearly half of the parents thought that antibiotics can be given to children with URTIs. This is concurrent with the study conducted in Malaysia [7]. This may be because of inadequate awareness creation in modern medicine and believe in traditional medicine. More than half of the parents believed that children with flu-like symptoms recovered faster when antibiotics were given. URTI is an infection of viral origin, so it should not be treated with antibiotics [26]. The emergence of bacterial resistant strains may rapidly develop due to the inappropriate use of antibiotics [27]. Antibiotics are used unnecessarily while more than one-third of them (39.5%) agreed that most of URTI are of viral origin. Thirty percent of the parents agreed that they may change their doctors because of prescribing antibiotics to their children to treat URTI. Slightly less than half of the parents (49.2%) agreed that most of URTI are self-limited.

Majority of Children (55.5%) with flu-like symptoms get better faster when antibiotics are given. Greater than three forth of (76.5%) of parents´ inappropriate use of antibiotics reduces their efficacy and leads to bacterial resistance. But regarding antibiotic use can prevent complications from Upper Respiratory Tract Infections in majority 59.6%. More than half of the parents (56.8%) sometimes agreed that medical doctor recommends antibiotics. Only 8.1% agree most of the times parents directly ask for their medical doctor to prescribe antibiotics. Very few parents 1.6% respond never respect the medical doctor´s instructions and advice. More than one third (39.1%) never insists on the medical doctor´s prescribing antibiotics as a precaution even if a diagnosis is not confirmed.

## Conclusion

In this study has documented many areas in which parental knowledge on antibiotic use for acute URTI is considered inadequate, consequently inappropriate knowledge and practices. Nearly half of the parents attending the physicians for their children with URTI expected to get antibiotics. Factors responsible for inappropriate use were a parental self-prescribing tendency, the parental tendency of asking for antibiotics from the doctor. Parents are self-prescribing because of their availability of antibiotics without prescription in pharmacies and their indifferent awareness toward microbial resistance. Parents pointed out that more advice is needed for both doctors and parents for proper antibiotic utilization for children.

### What is known about this topic

- Nearly half of the parents attending the physicians for their children with URTI expected to get antibiotics;
- Factors responsible for inappropriate use were a parental self-prescribing tendency, the parental tendency of asking for antibiotics from the doctor;
- Parents are self-prescribing because of their availability of antibiotics without prescription in pharmacies and their indifferent awareness toward microbial resistance.

### What this study adds

- It is important to introduce evidence-based clinical practice guidelines/manual of acute URTI for clinicians with a regular medical audit of treatment for acute URTI to ensure that patients receive the best quality of care;
- The parents and physicians should have a trusted relationship because most parents will be happy with the information;
- The regional health bureau should prepare guideline regarding the management of respiratory infection.

## Competing interests

The authors declare no competing interests.
